# Quantile-specific heritability of plasma fibrinogen concentrations

**DOI:** 10.1371/journal.pone.0262395

**Published:** 2022-01-07

**Authors:** Paul T. Williams

**Affiliations:** Lawrence Berkeley National Laboratory, Molecular Biophysics & Integrated Bioimaging Division, Berkeley, CA, United States of America; Cleveland Clinic Lerner Research Institute, UNITED STATES

## Abstract

**Background:**

Fibrinogen is a moderately heritable blood protein showing different genetic effects by sex, race, smoking status, pollution exposure, and disease status. These interactions may be explained in part by “quantile-dependent expressivity”, where the effect size of a genetic variant depends upon whether the phenotype (e.g. plasma fibrinogen concentration) is high or low relative to its distribution.

**Purpose:**

Determine whether fibrinogen heritability (*h*^*2*^) is quantile-specific, and whether quantile-specific *h*^*2*^ could account for fibrinogen gene-environment interactions.

**Methods:**

Plasma fibrinogen concentrations from 5689 offspring-parent pairs and 1932 sibships from the Framingham Heart Study were analyzed. Quantile-specific heritability from offspring-parent (β_OP_, *h*^*2*^ = 2β_OP_/(1+r_spouse_)) and full-sib regression slopes (β_FS_, *h*^*2*^ = {(1+8r_spouse_β_FS_)^0.05^–1}/(2r_spouse_)) were robustly estimated by quantile regression with nonparametric significance assigned from 1000 bootstrap samples.

**Results:**

Quantile-specific *h*^*2*^ (±SE) increased with increasing percentiles of the offspring’s age- and sex-adjusted fibrinogen distribution when estimated from β_OP_ (P_trend_ = 5.5x10^-6^): 0.30±0.05 at the 10^th^, 0.37±0.04 at the 25^th^, 0.48±0.05 at the 50^th^, 0.61±0.06 at the 75^th^, and 0.65±0.08 at the 90^th^ percentile, and when estimated from β_FS_ (P_trend_ = 0.008): 0.28±0.04 at the 10^th^, 0.31±0.04 at the 25^th^, 0.36±0.03 at the 50^th^, 0.41±0.05 at the 75^th^, and 0.50±0.06 at the 90^th^ percentile. The larger genetic effect at higher average fibrinogen concentrations may contribute to fibrinogen’s greater heritability in women than men and in Blacks than Whites, and greater increase from smoking and air pollution for the *FGB* -455G>A A-allele. It may also explain greater fibrinogen differences between: 1) *FGB* -455G>A genotypes during acute phase reactions than usual conditions, 2) *GTSM1* and IL-*6* -572C>G genotypes in smokers than nonsmokers, 3) *FGB* -148C>T genotypes in untreated than treated diabetics, and *LPL PvuII* genotypes in macroalbuminuric than normoalbuminuric patients.

**Conclusion:**

Fibrinogen heritability is quantile specific, which may explain or contribute to its gene-environment interactions. The analyses do not disprove the traditional gene-environment interpretations of these examples, rather quantile-dependent expressivity provides an alternative explanation that warrants consideration.

## Introduction

Elevated plasma fibrinogen is an independent risk factor for ischemic heart disease, stroke, other vascular mortality, and nonvascular mortality [1]. Fibrinogen also is a major determinant of plasma viscosity, precursor of fibrin, mediator of platelet aggregation [2], and stimulator smooth muscle cell migration and proliferation [3]. The accumulation of fibrinogen and fibrin in atherosclerotic plaque is proportional to fibrinogen concentrations in plasma [4]. Various factors are reported to elevate fibrinogen concentrations: age [5,6], female sex [5,6], menopause and oral contraceptives use [6], obesity [5–7], cigarette smoking [5,7], type 2 diabetes (T2DM) [6], low education and socioeconomic status [5], and traffic-related pollution [8]. Fibrinogen is also an acute-phase reactant that increases in response to inflammatory triggers such as infection, acute exercise and severe injury [9]. Alcohol and physical activity lower fibrinogen [5,7].

Fibrinogen is moderately heritable. Twin and family studies suggest that approximately 30% to 50% of the variability of fibrinogen concentrations is attributed to additive genetic effects [10], although estimates vary (21% [11], 23% [12], 24% [13], 27% [14], 30% [15–17], 31% [18]; 34% [19,20], 35% [21], 39% [22], 44% [23], 45% [16], 50% [24,25], 51% [26], 52% [27], 66% [28], and 67% [29]). Non-additive genetic effects are also suggested [17]. The fibrinogen protein is made up of two chains of each of three different polypeptides called alpha (Aα), beta (Bβ), and gamma polypeptide chains, which are encoded by three different genes: fibrinogen alpha chain (*FGA*), beta chain (*FGB*), and gamma chain (*FGG*), respectively [30]. Synthesis of the fibrinogen Bβ-chain is the rate-limiting step in the production of mature fibrinogen proteins [31]. Fibrinogen concentrations are higher for carriers of the G to A substitution in the 5’ flanking region of the *FGB* promoter at position −455 vis-à-vis the GG homozygotes (rs1800790, AKA *Hae*III H2-carriers and H1H1 genotype, respectively) [32]. Although this and other genetic variants within the Bβ-chain show the strongest effects on plasma fibrinogen concentrations, they still only explain about 1% of fibrinogen variation.

Traditional genetic analyses assume that the effect of a gene is a constant throughout its distribution. Alternatively, quantile-dependent expressivity occurs when the phenotypic expression of a gene depends upon the level of the phenotype, i.e., whether the trait (e.g., plasma fibrinogen concentration) is relatively high or low [33–41]. We have shown that the heritability of adiposity [33,34], lipoprotein cholesterol [33,35], triglyceride [33,38], and adiponectin concentrations [37], and usual coffee [40] and alcohol intake [41] are all quantile-dependent. Specifically, heritability estimated from their offspring-parent and full-sib regression slopes increased significantly from the lowest to the highest percentiles of each trait’s distribution. Moreover, within individuals, the genetic effects size of many triglyceride-related single nucleotide polymorphisms (SNP) change over time during postprandial lipemia, i.e., in relation to the changing average triglyceride concentrations following fat ingestion [39]. The hypothesis may be supported in part by Porcu et al.’s finding that differentially expressed genes may reflect disease-induced rather than disease-causing changes in the transcriptome [42].

If the heritability of plasma fibrinogen concentrations is quantile-specific, then this could contribute to some of the gene-environment interactions reported for fibrinogen. Specifically, subjects selected for characteristics that distinguish high vs. low concentrations (e.g., smoking vs. nonsmoking) would be expected to have different size genetic effects. We therefore sought to test whether estimated heritability of fibrinogen concentrations from offspring-parent pairs and full sibs were quantile-specific in a large population cohort (Framingham Heart Study [43,44]). Heritability was analyzed because 47 independently significant variants at 41 independent loci collectively explained only about 3% of the variance of fibrinogen concentrations [45], i.e. a very small percentage of fibrinogen’s heritability. We also re-assessed published reports of fibrinogen gene-environment interactions from the perspective of quantile-dependent expressivity. The results suggest that quantile-dependent expressivity: 1) may contribute to the greater fibrinogen heritability in women than men [46] and in Blacks than Whites [47], and 2) is consistent with the genotype-specific effects of smoking [7,48–52], air pollution [53,54], coronary artery disease [49], acute-phase response [32,55–57], T2DM treatment [58], and albuminuria [59] on fibrinogen concentrations.

## Methods

The data were obtained from the National Institutes of Health FRAMCOHORT, GEN3, FRAMOFFSPRING Research Materials obtained from the National Heart Lung and Blood Institute (NHLBI) Biologic Specimen and Data Repository Information Coordinating Center. The hypothesis tested are exploratory and not considered as part of the initial Framingham Study design.

Subjects were at least 16 years of age and not self-identified as nonwhite or Hispanic. Fibrinogen concentrations were measured for examinations 5,6, and 7 of the Offspring Cohort, and examination 1 of the Third Generation Cohort. Venous blood samples were drawn in the morning into a 3.8% sodium citrate solution with a blood/sodium citrate ratio of 9:1 in volume, which was then centrifugation at 2000xg to produce platelet-poor plasma that was stored at -80°C. Plasma fibrinogen concentrations were measured by the Clauss method (Diagnostica Stago Reagents) [60], which showed intra-assay and inter-assay coefficients of variation of 2.6% and 4.7%, respectively.

Our analyses of these data were approved by Lawrence Berkeley National Laboratory Human Subjects Committee (HSC) for protocol “Gene-environment interaction vs. quantile-dependent penetrance of established SNPs (107H021)”. LBNL holds the Office of Human Research Protections Federal wide Assurance number FWA 00006253. The protocol has approval number: 107H021-13MR20. All surveys were conducted under the direction of the Framingham Heart Study human use committee guidelines, with signed informed consent from all participants or parent and/or legal guardian if <18 years of age.

### Statistics

The statistics are identical to those previously described [34–36,38]. Age and sex adjustment was performed using standard least-squares regression with the following independent variables: female (0,1), age, age^2^, female x age, and female x age^2^. Full-sibling correlations and regression coefficients (β_FS_) were obtained by constructing all possible pairs using double entry [61], with an adjusted Σ(k_i_-1) degrees of freedom, where k_i_ is the number of offspring in family i and the summation is taken over all i, i = 1,…, N nuclear families. Untransformed fibrinogen concentrations were analyzed because quantile regression does not require normality [62,63], and no biological justification has yet been given for its logarithmic transformation. Some reports on fibrinogen heritability and gene-environment interactions use untransformed fibrinogen concentrations [7,12,15,17,19,22,26,28,29,46,51,54,56–58], others used log-transformed values [14,16,20,21,23–25,27,47,49,52,53,59].

Simultaneous quantile regression was performed using the “sqreg” command of Stata (version. 11, StataCorp, College Station, TX) with one thousand bootstrap samples drawn to estimate the variance-covariance matrix for the 91 quantile regression coefficients (β_FS_) between the 5^th^ and 95^th^ percentiles, and the post-estimation procedures (test and lincom) to test linear combinations of the β_FS_ slopes after estimation with Σ(k_i_-1) degrees of freedom. Quantile-specific offspring-parent concordance was assessed by: 1) estimating quantile-specific β_OP_-coefficient for the 5^th^, 6^th^, …, 95^th^ percentiles of the sample distribution using simultaneous quantile regression (the <5^th^ and >95^th^ percentiles ignored because they were thought to be less stable); 2) plotting the quantile-specific β_OP_ coefficients vs. the percentile of the trait distribution; and 3) testing whether the resulting graph is constant, or changes as a linear, quadratic, or cubic function of the percentile of the trait distribution using orthogonal polynomials [64].

Heritability in the narrow sense (*h*^*2*^) was estimated as *h*^*2*^ = 2β_OP_/(1+r_spouse_) from offspring-parent regression slopes (β_OP_) and *h*^*2*^ = {(1+8r_spouse_β_FS_)^0.5^–1}/2r_spouse_ from full-sibs regression slopes (β_FS_) where r_spouse_ is the spouse correlation [65]. “Quantile-specific heritability” refers to the heritability statistic (*h*^*2*^), whereas “quantile-dependent expressivity” is the biological phenomenon of the trait expression being quantile-dependent. In some cases, published reports of gene-environment interactions were originally presented as histograms or line graphs [51,52,55,66], which were imported into Microsoft PowerPoint (version 12.3.6 for Macintosh computers, Microsoft corporation, Redmond WA) to extract their quantitative information as previously described [39]. Means, slopes, and *h*^*2*^ are presented with their standard errors (±SE).

Data availability: The data are not being published in accordance with the data use agreement between the NIH National Heart Lung, and Blood Institute and Lawrence Berkeley National Laboratory. However, the data that support the findings of this study are available from NIH National Heart Lung, and Blood Institute Biologic Specimen and Data Repository Information Coordinating Center directly through the website https://biolincc.nhlbi.nih.gov/my/submitted/request/ [67]. Restrictions apply to the availability of these data, which were used under license for this study. Those wishing a copy of the data set should contact the Blood Institute Biologic Specimen and Data Repository Information Coordinating Center at the above website, where they can find information on human use approval and data use agreement requiring signature by an official with signing authority for their institute. The public summary-level phenotype data may be browsed at the dbGaP study home page [68].

## Results

Offspring-parent regression analyses were performed on 1551 offspring with one parent and 2069 with both parents. Full-sib regression analysis were performed using 2045 siblings in 778 sibships from the Offspring Cohort, and 3334 siblings from 1154 sibships from the Third Generation Cohort. The characteristics for the participants used in the analyses are presented in Table 1. As expected, average plasma fibrinogen concentrations were higher for females than males in both cohorts.

**Table 1:** Sample characteristics.

	Offspring Cohort		Third Generation Cohort	
	Male	Female	Male	Female
N	1598	1798	1834	2046
Age years*	58.04 (9.72)	57.89 (9.67)	40.39 (8.73)	39.98 (8.77)
Fibrinogen g/L*	3.359 (0.577)	3.446 (0.593)	3.339 (0.677)	3.470 (0.729)

* mean (SD).

### Traditional estimates of familial concordance and heritability

Spouses exhibited modest similarity (r_spouse_ = 0.143) as reported by others [17,22]. The offspring-parent regression slope for age- and sex-adjusted fibrinogen was β_OP_ = 0.272±0.015, which corresponds to an estimated heritability of *h*^*2*^ = 0.475±0.035 (P<10^−16^), consistent with published values. The heritability was greater for female than male offspring (*h*^*2*^: 0.576±0.049 vs. 0.358±0.049). Heritability estimated from the offspring-midparental slope was 0.416±0.034 (P<10^−16^, *h*^*2*^ = β_OM_). The full-sib regression slope (β_FS_ = 0.188±0.017, P<10^−16^) and corresponding heritability estimate (*h*^*2*^ = 0.358±0.033) were somewhat less than their offspring-parent derived values.

### Quantile-dependent expressivity

**Fig 1:**
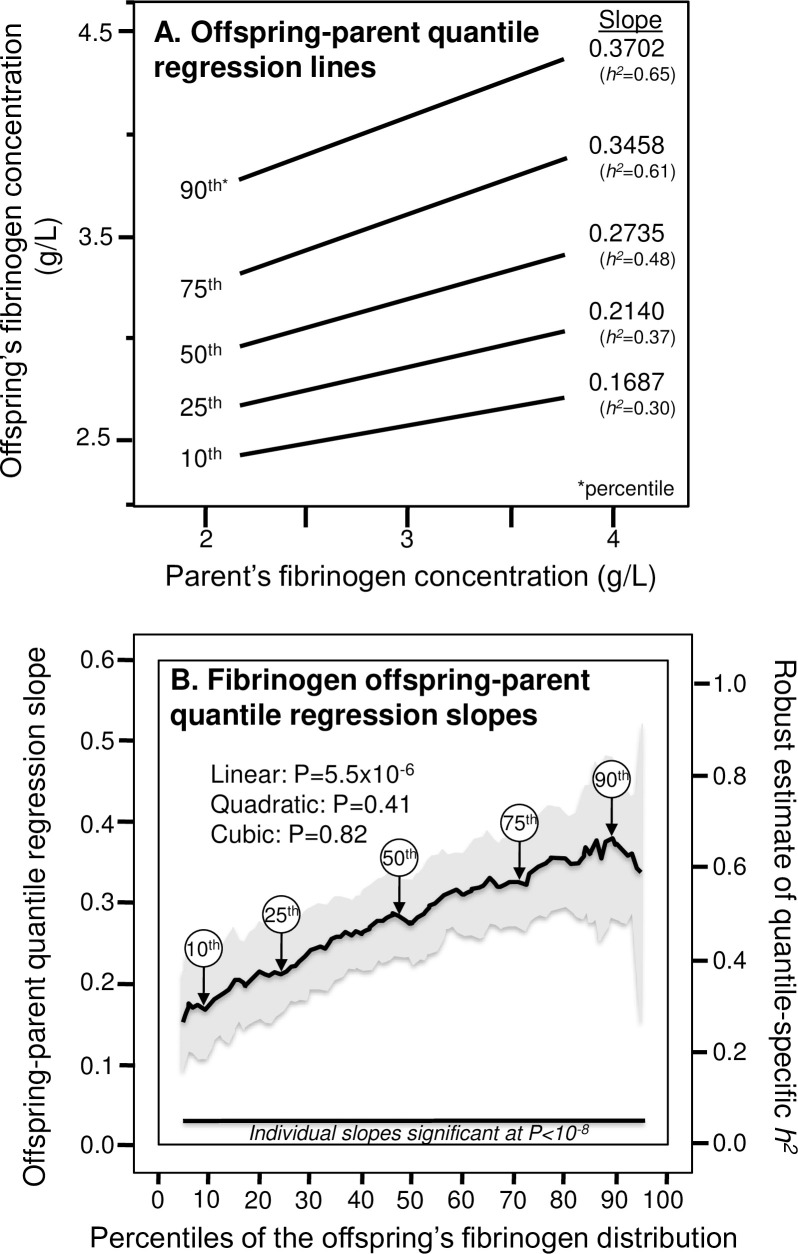
Quantile regression analysis of offspring-parent pairs. (A) Offspring-parent regression slopes (β_OP_) for selected quantiles of the offspring’s fibrinogen concentrations from 7769 offspring-parent pairs, with corresponding estimates of heritability (*h*^*2*^ = 2β_OP_/(1+r_spouse_) [65], where the correlation between spouses was r_spouse_ = 0.168. The slopes became progressively greater (i.e., steeper) with increasing quantiles of the fibrinogen distribution. (B) The selected quantile-specific regression slopes were included with those of other quantiles to create the quantile-specific heritability function in the lower panel. Significance of the linear, quadratic and cubic trends and the 95% confidence intervals (shaded region) determined by 1000 bootstrap samples.

Fig 1A presents the offspring-parent regression slopes (β_OP_) at the 10^th^, 25^th^, 50^th^, 75^th^, and 90^th^ percentiles of the offspring’s age- and sex-adjusted fibrinogen distribution. The slopes, and their corresponding heritability estimates (*h*^*2*^ = 2*β_OP_/(1+r_spouse_)), get progressively steeper with increasing percentiles of the distribution. The heritability at the 90^th^ percentile was 2.2-fold greater than the heritability at the 10^th^ percentile (P_difference_ = 5.6x10^-5^). Specifically, the figure shows that *h*^*2*^ increased from 0.295±0.047 at their 10th percentile (P = 4.5x10^-10^), 0.374±0.040 at the 25th (P<10^−15^), 0.479±0.045 at the 50th (P<10^−15^), 0.605±0.056 at the 75th (P<10^−15^), and 0.648±0.082 at the 90th percentile of the offspring’ distribution (P<3.3x10^-15^). These slopes, along with those of the other percentiles between the 5^th^ and 95^th^ percentiles are presented in the quantile-specific heritability plot in Fig 1B. The corresponding *h*^*2*^ are displayed on the right. They show heritability increased linearly with increasing percentiles of the offspring’s distribution (i.e., slope±SE: 0.0040±0.0009 for each percent increment, P_linear_ = 5.5x10^-6^) with no significant evidence of nonlinearity (i.e., P_quadratic_ = 0.41; P_cubic_ = 0.82). Quantile-specific heritability was significant (P≤1.8x10^-6^) for all 91 individual percentiles between the 5^th^ and 95^th^ percentiles of the offspring’s distribution. If the classical model of constant heritability over all quantiles applied, then the line segments in Fig 1A would be parallel, and the graph in Fig 1B would be flat (i.e., zero slope). Heritability estimated from the offspring-midparental slope also increased linearly with increasing percentiles of the fibrinogen distribution (0.0033±0.0009, P_linear_ = 0.0005, not displayed).

**Fig 2:**
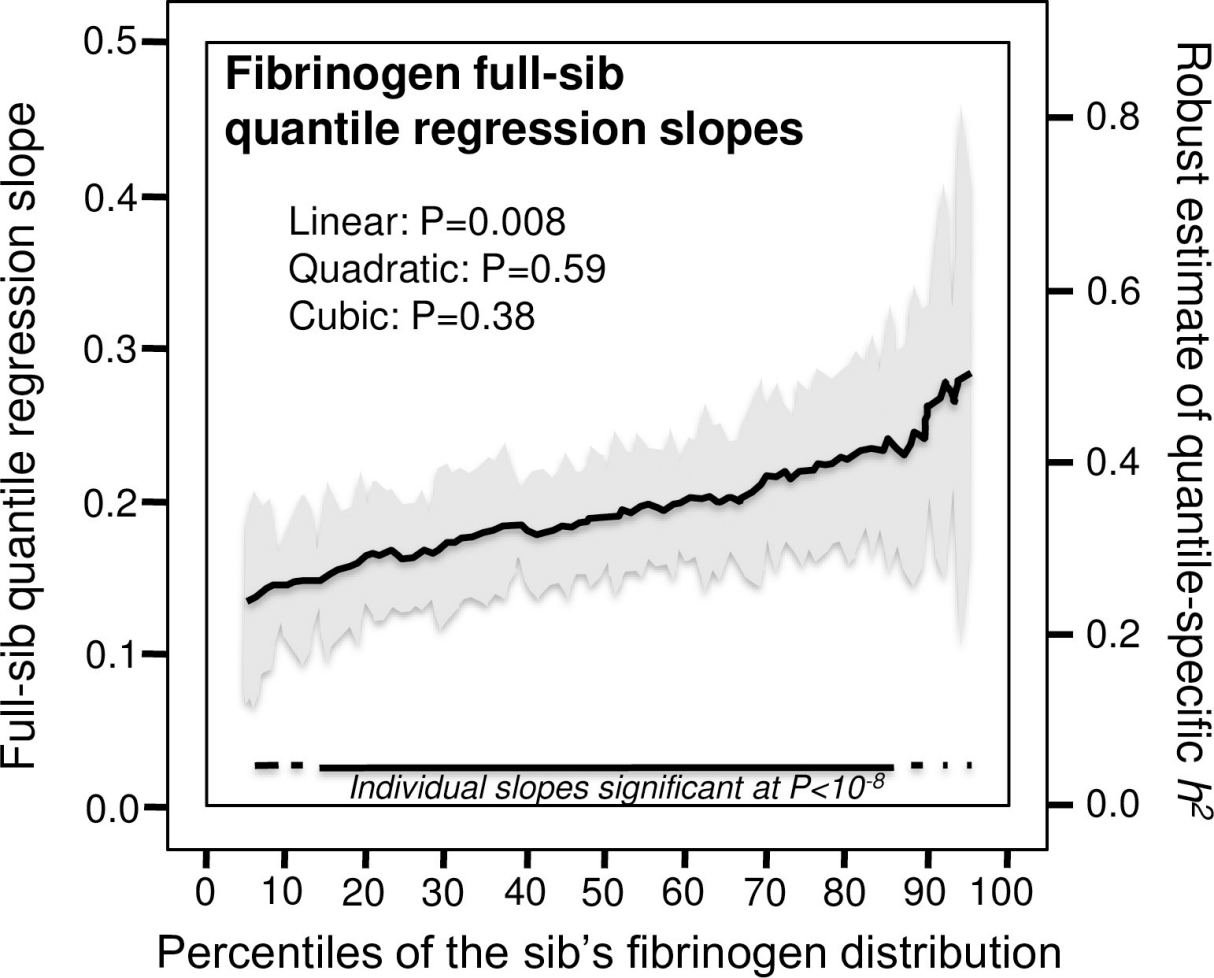
Quantile regression analysis of full-sib pairs. Quantile-specific full-sib regression slopes (β_FS_) from 5379 siblings in 1932 sibships, with corresponding estimates of heritability as estimated by *h*^*2*^ = {(8r_spouse_β_FS_+1)^0.5^–1}/(2r_spouse_) [65].

Fig 2 displays the quantile regression analysis for *h*^*2*^ estimated from full-sib regression slopes (β_FS_). Each one-percent increase in the fibrinogen distribution was associated with a 0.0026±0.0010 increase in heritability and a 0.0013±0.0005 increase in the full-sib regression slope (P_linear_ = 0.008).

## Discussion

### Gene-environment interaction

Quantile-dependent expressivity may contribute to gene-environment interactions reported for plasma fibrinogen concentrations. Specifically, the selection of subjects by characteristics that distinguish high from low fibrinogen concentrations (e.g., smokers vs. nonsmokers, exposed vs. unexposed to pollution, untreated vs. treated T2DM) is expected to create different genetic effects that might be simply explained by quantile-specific heritability (Fig 1). The examples that follow present genotype-specific effects of environmental factors on fibrinogen concentrations (a precision medicine interpretation) that quantile-dependent expressivity would attribute to a larger genetic effect size at higher average fibrinogen concentrations. The analyses do not disprove the traditional gene-environment explanation these interactions, rather quantile-dependent expressivity provides an alternative explanation that warrants consideration.

### Sex and ethnicity

For example, meta-analyses suggest that fibrinogen concentrations are 0.16 g/L higher in women than in men and 0.12 g/L higher in Blacks than Whites [1,5]. Friedlander et al. [46] reported significantly larger genetic standard deviations in females than males, and correspondingly, greater female heritability (56% and 23%). Ding et al. [47] reported that fibrinogen heritability was greater in African-American than Non-Hispanic Whites (44% vs. 28%) in accordance with their higher average fibrinogen concentrations (2.95±0.01 vs. 1.52±0.01 g/L, P<0.0001). Some of these sex and racial difference could be the consequence of quantile-dependent expressivity, i.e., larger genetic effect size at higher concentrations.

### Smoking

Smoke-induced inflammations of the lungs and other organs increase proinflammatory cytokines and circulating inflammatory markers [5]. Baumert et al.’s meta-analysis of 80,607 participants of European ancestry showed that current smokers had 0.163 g/L higher average fibrinogen concentrations than nonsmokers [7].

**Fig 3:**
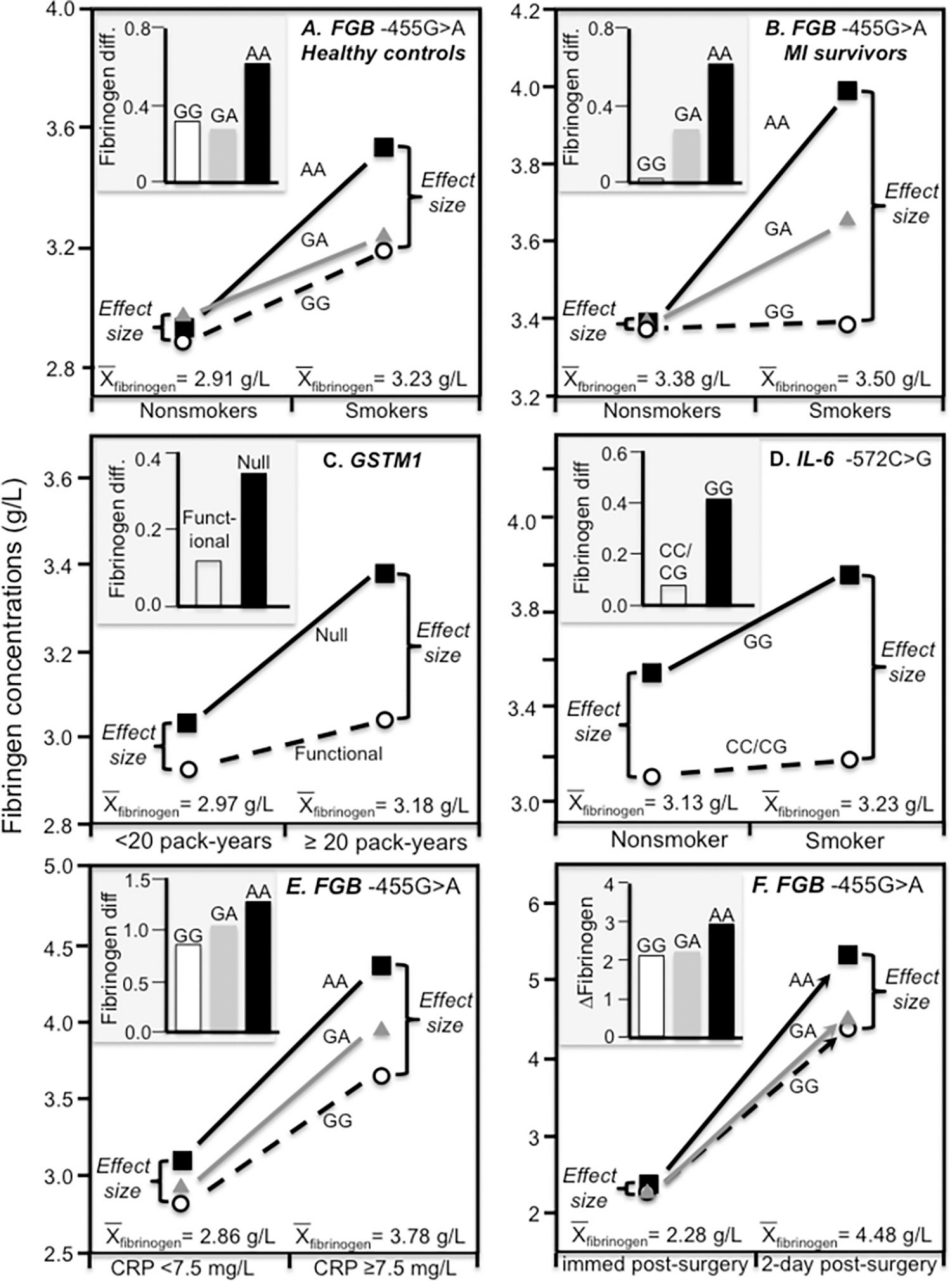
Precision medicine perspective of genotype-specific fibrinogen differences (histogram inserts) vs. quantile-dependent expressivity perspective (line graphs showing larger genetic effect size when average fibrinogen concentrations were high) for: (A) Scarabin et al.’s 1993 report [50] on the smokers-nonsmoker difference by the −455G>A polymorphism of the fibrinogen beta chain (*FGB*) promoter region in healthy controls; (B) Scarabin et al.’s 1993 report [50] on the aforementioned smokers-nonsmoker difference in myocardial infarction survivors; (C) Miller et al.’s 2003 report [51] on the difference between more vs. less pack-years of smoking by the glutathione-s-transferases (GTS) M1 polymorphism; (D) Shin et al.’s 2007 report [52] on the smokers-nonsmoker difference by the interleukin-6 (*IL6*) -572C>G polymorphism; (E) Gardemann et. al.’s 1997 report [55] on the difference between patients with clinical signs of an acute phase reaction (i.e., CRP≥ 50^th^ percentile) than without by FGB −455G>A polymorphism; (F) Gardemann et. al.’s 1997 report [55] on the fibrinogen increase 2-days after aortacoronary bypass surgery by the FGB −455G>A polymorphism.

Initial reports of smoking-gene interactions for the *FGB* -455G>A rs1800790 polymorphism gave conflicting results. Whereas Green et al. [48] reported that the polymorphism affected fibrinogen concentrations only the smokers, Thomas et al. [69] reported that the polymorphism’s effect was limited to nonsmokers. However, both studies were small, and Baumert et al.’s analysis of a much larger sample [7] confirmed the association in smokers but not nonsmokers, consistent with the smokers’ higher average fibrinogen concentrations. In the Etude Cas-Temoins sur l’Infarctus du Myocarde (ECTIM) Study, Behague et al. reported that the minor allele produced a significantly greater effect on log plasma fibrinogen concentrations (P_interaction_<0.05) in smokers (partial r^2^ = 0.0239 per copy, P<0.0004) than nonsmokers (partial r^2^ = 0.0028 per copy, NS) [49], corresponding to the smokers’ higher average concentrations [50]. Fig 3A and 3B present results for myocardial infarction survivors and controls separately [50], showing the genotype-specific effects of smoking (histograms) could be attributed in part to the larger genetic effect size at the smokers’ higher average fibrinogen concentrations. The authors also reported that the polymorphism interacted significantly with number of cigarettes smoked per day in affecting concentrations (P<0.03) [49], consistent with the dose-related trend between fibrinogen concentrations and daily cigarette use [5].

Glutathione-S-transferases (GTS) are phase II enzymes that catalyze glutathione’s reaction with dichloromethane and polycyclic aromatic hydrocarbons in cigarette smoke to produce inactive and non-toxic hydrophilic conjugates. Miller et al. [51] reported that the affect of smoking more vs. less than 20 pack-years on plasma fibrinogen concentrations was greater for the M1-0 (null) than M1-1 (functional) genotypes of the GTSM1 polymorphism (Fig 3C, P_interaction_ = 0.06). However, average fibrinogen concentrations were significantly greater in the ≥ 20 pack-year smokers (3.18 vs. 2.97 g/L) and quantile-dependent expressivity would attribute some of the interaction to the larger genetic effect size (0.335 vs. 0.106 mg/dL) at the higher average fibrinogen concentrations of the more-exposed smokers, i.e., a greater impact of not detoxifying toxins at higher fibrinogen concentrations [51].

Interleukin-6 (IL-6) is an important upstream messenger cytokine in inflammation that regulates hepatic fibrinogen production by stimulating *FGA*, *FGB*, and *FGG* promoter regions. It is the primary mediator of acute phase-induced fibrinogen synthesis [70]. The IL-6 -572C>G polymorphism is a relatively common allele in Koreans affecting transcription strength. Results presented by Shin et al. [52] (their Fig 2) suggest a somewhat greater effect of smoking on the average fibrinogen concentrations in GG homozygotes than carriers of the C allele (Fig 3D histogram) which quantile-dependent expressivity would attribute to a greater effect of the -572C>G polymorphism at higher fibrinogen concentrations (Fig 3D line graph).

### Pollution

Fibrinogen concentrations are higher after exposure to traffic pollutants [8]. This may be due to particles deposited in the lungs inducing alveolar inflammation and the cytokine release causing systemic inflammation. Bind et al. [53] reported that the percent change in fibrinogen concentrations associated with air pollution particle number was significantly greater for a high vs. low genetic risk score (P = 0.04). Peters et al. [54] reported that the increase in fibrinogen concentrations with increasing PM_10_ concentrations (i.e., particulate matter with an aerodynamic diameter <10 μm) was 8-fold greater in -455 AA homozygotes than GG homozygotes (P_interaction_ = 0.04). The authors interpreted their results from the perspective of genotype-specific susceptibility to ambient particulate matter, where the risk alleles produced an augmented response to environmental inflammatory stimuli in addition to constitutionally higher fibrinogen concentrations. The risk alleles were hypothesized to affect early physiological responses such fibrinogen transcription. Alternatively, an interpretation from the perspective of quantile-dependent expressivity would argue that environmental inflammatory stimuli increase average fibrinogen concentration, which in turn, accentuated the genetic effects. Instead of being a susceptibility locus, *FGB* -455G>A may simply track the increasing heritability at higher fibrinogen concentrations.

### Acute-phase response

**Fig 4:**
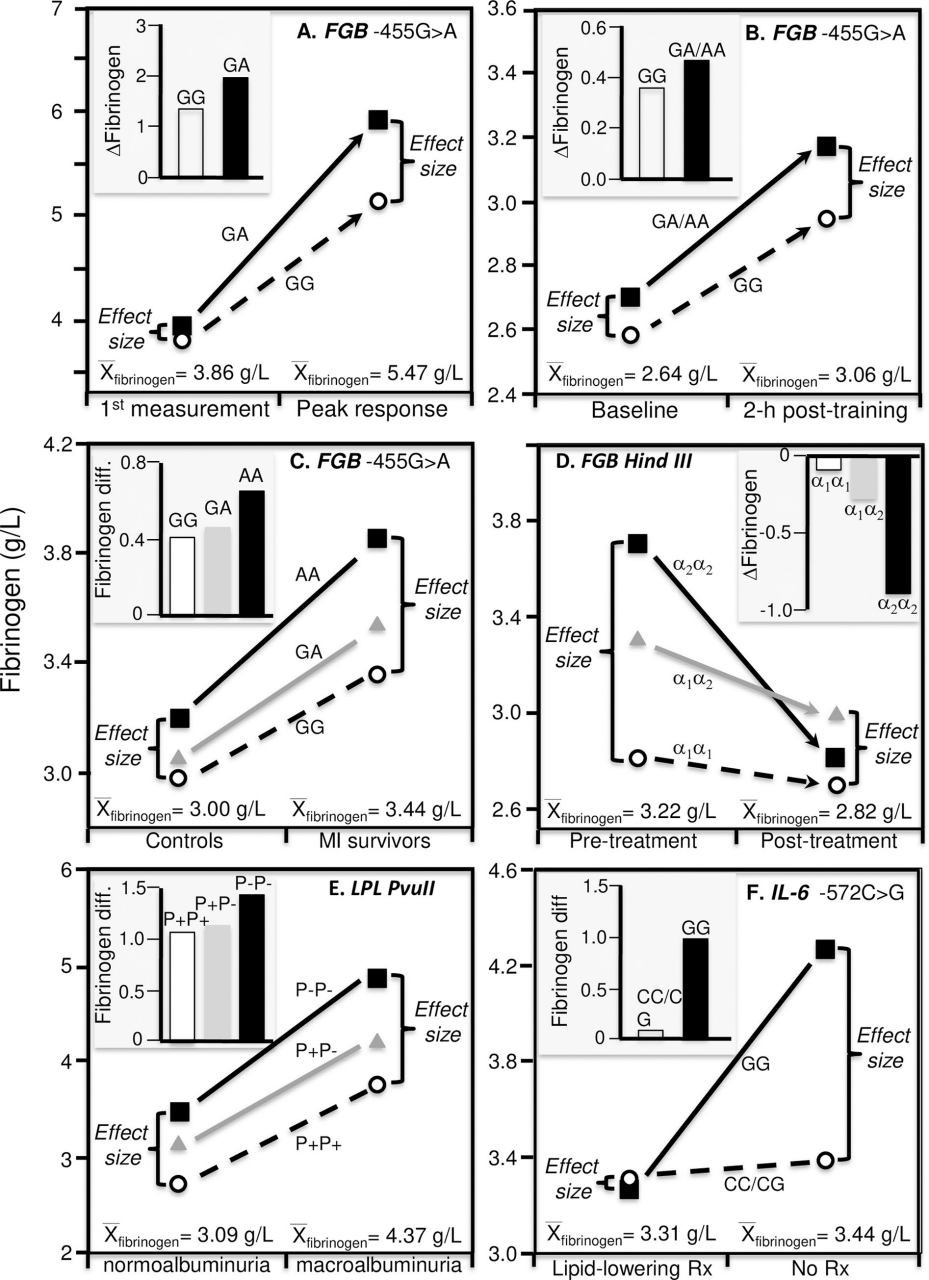
Precision medicine perspective of genotype-specific fibrinogen differences (histogram inserts) vs. quantile-dependent expressivity perspective (line graphs showing larger genetic effect size when average fibrinogen concentrations were high) for: (A) Ferrer-Antunes 1998 report [56] on increases in fibrinogen concentrations during the peak acute phase response following acute cranial or thoracic trauma by −455G>A genotypes; (B) Brull et al.’s 2002 report on the changes in fibrinogen concentrations due to two days strenuous military exercise by the −455G>A polymorphism [57]; (C) Behague et al.’s 1996 report [49] on the difference by myocardial infarction (MI) survivors and healthy controls by the *FGB* -455G/A polymorphism; (D) Ceriello et al. 1998 report [58] on changes in fibrinogen concentration from intensive insulin therapy in T2DM patients under poor metabolic control by *FGB* -148C>T polymorphism; (E) Javorský et al. 2006 report [59] on the difference between macroalbuminia and normoalbuminuria patients by the lipoprotein lipase (*LPL) PvuII* polymorphism; (F) Jang et al.’s 2008 report [66] on differences between coronary heart disease patients on and off lipid lowering medications (Rx) by IL-6 -572C>G genotypes.

Increases the hepatic synthesis and release of fibrinogen and other acute-phase reactants may be triggered by specific inflammatory cytokine signals following severe injury or exercise recovery [71]. C-reactive protein (CRP) is both an acute-phase protein and a general marker for inflammation. Gardemann et al. reported that the difference between *FGB* -455 AA and GG homozygotes was almost three-fold greater in subjects with clinical signs of an acute phase reaction (i.e., a CRP≥ the 50^th^ percentile) than without, consistent with the higher mean fibrinogen concentrations during the reaction (Fig 3E) [55]. They also reported that AA-homozygotes showed significantly greater fibrinogen increases 2-days after aortacoronary bypass surgery than carriers of the G-allele (Fig 3F histogram), which the line graph suggests could be simply the consequence of the higher fibrinogen concentrations two days post-surgery. Another study by Ferrer-Antunes et al. [56] concluded that the increase of fibrinogen level in acute phase situations like severe trauma is associated with the β-gene -455G/A polymorphism (Fig 4A histogram). Again, the line graph shows that following acute cranial or thoracic trauma, the differences between -455 A-allele carriers vs. GG homozygotes were greater at peak concentrations (0.77±0.34 g/L) when average fibrinogen concentrations were highest (5.47±0.17 g/L) than when first measured (effect size 0.14±0.40 g/L) when average concentrations were lower (3.86±0.20 g/L).

Brull et al. reported that a 2-day strenuous military exercise produced greater acute-phase fibrinogen increases in *FGB* -455 A-allele carriers than GG homozygotes at 2 hours (0.23±0.07 g/L genotype difference, P<0.005, Fig 4B), 48 hours (0.26±0.07 g/L difference, P<0.0005), and 96 hours after training (0.26±0.09 g/L difference, P<0.0005) as compared to baseline (0.12±0.07 g/L difference, P = 0.11), corresponding to the higher average fibrinogen concentrations at 2 hours (3.06±0.03 g/L), 48 hours (2.73±0.03 g/L), and 96 hours after training (2.84±0.04 g/L) as compared to baseline (2.64±0.04 g/L) [57]. This followed an earlier report by Humphries et al. [32] of increased plasma fibrinogen concentrations 1 to 3 days after the two-day strenuous military exercise, during which the fibrinogen concentrations rose 27% for the GG, 37% for the GA, and 89% for the AA genotypes, i.e., the genetic differences were accentuated at the higher post-exercise fibrinogen concentrations.

### Coronary heart disease

In the ECTIM Study, Behague et al. [49] reported that the affect of the *FGB* -455G/A polymorphism on plasma fibrinogen concentrations appeared to be stronger in cases having a prior history of definite acute MI than those without a history, particularly in smokers [50], consistent with the cases’ higher average fibrinogen concentrations (3.44±0.04 vs. 3.00±0.03 g/L, Fig 4C).

### Diabetes

Ceriello et al. [58] reported that intensive insulin therapy in T2DM patients under poor metabolic control significantly reduced fibrinogen concentrations in the α_2_α_2_ and α_1_α_2_ genotypes (P<0.001) of the *Hind* III polymorphism in the *FGB* 5’ region (AKA -148C>T, in complete allelic association with -455G>A [32]) but not the α_1_α_1_ genotype (P_interaction_<0.001), which they attributed to a relationship between the genotypes and glycaemic control (Fig 4D histogram). Fibrinogen levels were significantly higher before than after treatment (3.22 vs. 2.82 g/L), and an interpretation from the perspective of quantile-dependent expressivity attributes the results to the larger genetic effect before treatment (0.45 g/L per α_2_-allele) when fibrinogen concentrations were high vs. after treatment (0.05 g/L per α_2_-allele) when fibrinogen concentrations were lower [58].

### Albuminuria

A strong correlation between urinary albumin excretion rate and fibrinogen plasma levels reported by Javorský et al. in T2DM patients (r = 0.48, p = 0.001) may reflect their common relationship with underlying inflammation [59]. The histogram in Fig 4E shows progressively increasing fibrinogen difference between macro and normoalbuminuria by the number of P- alleles of the lipoprotein lipase (*LPL*) *PvuII* polymorphism. However, the line graph shows that while average fibrinogen concentrations increased from 3.09 g/L in normoalbuminuria (urinary albumin excretion <20 mg/L), to 3.47 g/L in microalbuminuria (20–199 mg/L), to 4.37 g/L in macroalbuminuria (≥200 mg/L), the fibrinogen difference between P-P- and P+P+ homozygotes was greater for macroalbuminia (1.11 g/L) than for microalbuminuria (0.53 g/L) or normoalbuminuria (0.75 g/L) [59], consistent with quantile-dependent expressivity.

### Lipid-lowering drugs

Jang et al. [66] reported that fibrinogen concentrations were significantly higher in coronary heart disease patients who were not taking lipid-lowering drugs than those that were (>80% statins). Whereas the authors concluded that lipid-lowering drugs prevented the fibrinogen-raising effect of the GG genotype of the IL-6 -572C>G polymorphisms (Fig 4F histogram), quantile-dependent expressivity suggests that the higher average fibrinogen concentrations of the untreated patients (line graph) could have contributed to the interaction.

### Caveats and limitations

None of the SNPs identified to date explain any more than a few percent of fibrinogen heritability, which means that the effects of any particular SNP is not necessarily constrained by the results of Fig 1. Humphries et al. report [32] of a diminishing (not increasing) affect of the *FGB* -455G>A polymorphism with age-related increases in men’s average fibrinogen concentrations, and Rauramaa et al.’s [72] report of larger (not smaller) fibrinogen differences between *FGA* RsaI genotypes in the most physically active women with the lower average concentration vis-à-vis less-active women with higher average concentrations, are examples of environmental effect that are potentially inconsistent with quantile-dependent expressivity. We also acknowledge that the simple estimates of *h*^*2*^ from Falconer formula [65] probably do not adequately describe fibrinogen inheritance, i.e., those derived from β_OP_ may include shared environmental effects, and those derived from β_FS_ may include shared environment and dominance effects and unmet restrictions on assortative mating. Finally, although there are multiple reports of interactions between the FGB -455G>A polymorphism and smoking, other reported gene-environment interaction generally lack replication.

## Conclusion

Our analyses of the Framingham Heart Study cohorts suggest quantile-specific heritability of plasma fibrinogen concentrations. This was obtained by applying quantile regression to both offspring-parent and full-sib age- and sex-adjusted values. Heritability at the 90^th^ percentile of the fibrinogen distribution (0.65±0.08) was 2.2-fold greater than the heritability at the 10^th^ percentile (0.30±0.05). Quantile-dependent expressivity, and the selection of subjects from different portions of the fibrinogen distribution, potentially provides an alternative explanation for many gene-environment interactions reported for fibrinogen.
